# Prognostic and clinicopathological significance of microRNA-494 overexpression in cancers: a meta-analysis

**DOI:** 10.18632/oncotarget.22633

**Published:** 2017-11-03

**Authors:** Zhenxian Xiang, Min Sun, Zewei Yuan, Chunxiao Zhang, Jun Jiang, Sihao Huang, Bin Xiong

**Affiliations:** ^1^ Department of Oncology, Zhongnan Hospital of Wuhan University, Hubei Key Laboratory of Tumor Biological Behaviors and Hubei Cancer Clinical Study Center, Wuhan 430071, P. R. China; ^2^ Department of General Surgery, Taihe Hospital, Hubei University of Medicine, Shiyan 442000, P. R. China

**Keywords:** meta-analysis, biomarker, hsa-miR-494, cancer, prognosis

## Abstract

MicroRNA-494 was revealed as an attractive prognostic biomarker in recent studies. Nevertheless, the prognostic value of microRNA-494 in cancers remains controversial. Current meta-analysis aims to elucidate the precise predictive value of microRNA-494 in various cancers. Eligible studies were identified through multiple search strategies, the hazard ratios (HRs) and their confidence interval (CI) for patient prognostic outcomes were extracted and estimated. The pooled results of fifteen studies indicated that elevated expression of microRNA-494 implies a good overall survival of cancer patients (HR = 0.58, 95% CI: 0.36–0.91); While no significant association was found between the high expression of microRNA-494 and clinicopathological characteristic. Additionally, subgroup analysis revealed that overexpression of microRNA-494 predicted a worse overall survival in non-small cell lung cancer (HR = 2.35, 95% CI: 1.05–5.24) and colorectal cancer (HR = 2.59, 95% CI: 1.62–4.14). As per the subgroup analysis, the cancer type, the anatomy system classification and the ethnic background had influence on the overall survival result. Our findings indicate that elevated expression of microRNA-494 might predict a good overall survival in most cancers, while in non-small cell lung cancer and colorectal cancer, overexpression of microRNA-494 might predict a worse overall survival.

## INTRODUCTION

Due to population growth and aging, as well as increased adoption of unhealthy lifestyle such as drinking, smoking and “westernized” diets, cancer has become a serious problem of public health throughout the planet [1]. The 5-year relative survival rates for all cancers were 37% among the Chinese and 69% for Americans [2, 3]. Therefore, it is essentially necessary to identify valuable molecular biomarkers to promote early detection, prognostic classification, and novel therapeutic for cancers.

MicroRNAs (miRNAs), which are approximately 18–25 nucleotides in length, bind to complementary sequences of mRNA at the 3^’^-untranslated region and lead to mRNA degradation or translational repression [4, 5]. From the first discovery of miRNAs in 1993 to the present day [6], plenty of studies demonstrated that miRNAs participate in numerous biological processes, which include cellular growth, metabolism, differentiation, proliferation, apoptosis, and angiogenesis [7, 8]. Moreover, recent studies had demonstrated that many miRNAs expressed aberrantly in various kinds of cancers, which were further demonstrated to be related to cancer development, progression, and especially carcinogenic treatment [9–12]. Therefore, these miRNAs hold great promise for predicting the cancer patients’ prognosis.

Recently, many studies have demonstrated that microRNAs have potential prognostic value. As a gene located on q32-q31 of the fourteenth human chromosome’s long arm [13], microRNA-494 (miR-494, miRNA-494) has been reported to be differentially expressed in various cancers. In Acute Myeloid Leukemia (AML), it has been observed that overexpressed miR-494 was associated with good prognosis [14]. Up to now, overexpressed miR-494 has been reported to be related to good survival in head and neck squamous cell carcinomas (HNSCC) [15], nasopharyngeal carcinoma (NPC) [16], gastric carcinoma (GC) [17], pancreatic carcinoma (PC) [18–20], ovarian cancer (OC) [21], cervical cancer (CC) [22], as well as chondrosarcoma [23]. Meanwhile, there are investigators who get insignificant or even opposite results [24–27]. Therefore, we conducted this meta-analysis, through which we could get all the published articles of miR-494 to comprehensively evaluate the effect of miR-494 on the prognosis of cancer patients.

## RESULTS

### Summary of the included studies

**Figure 1:**
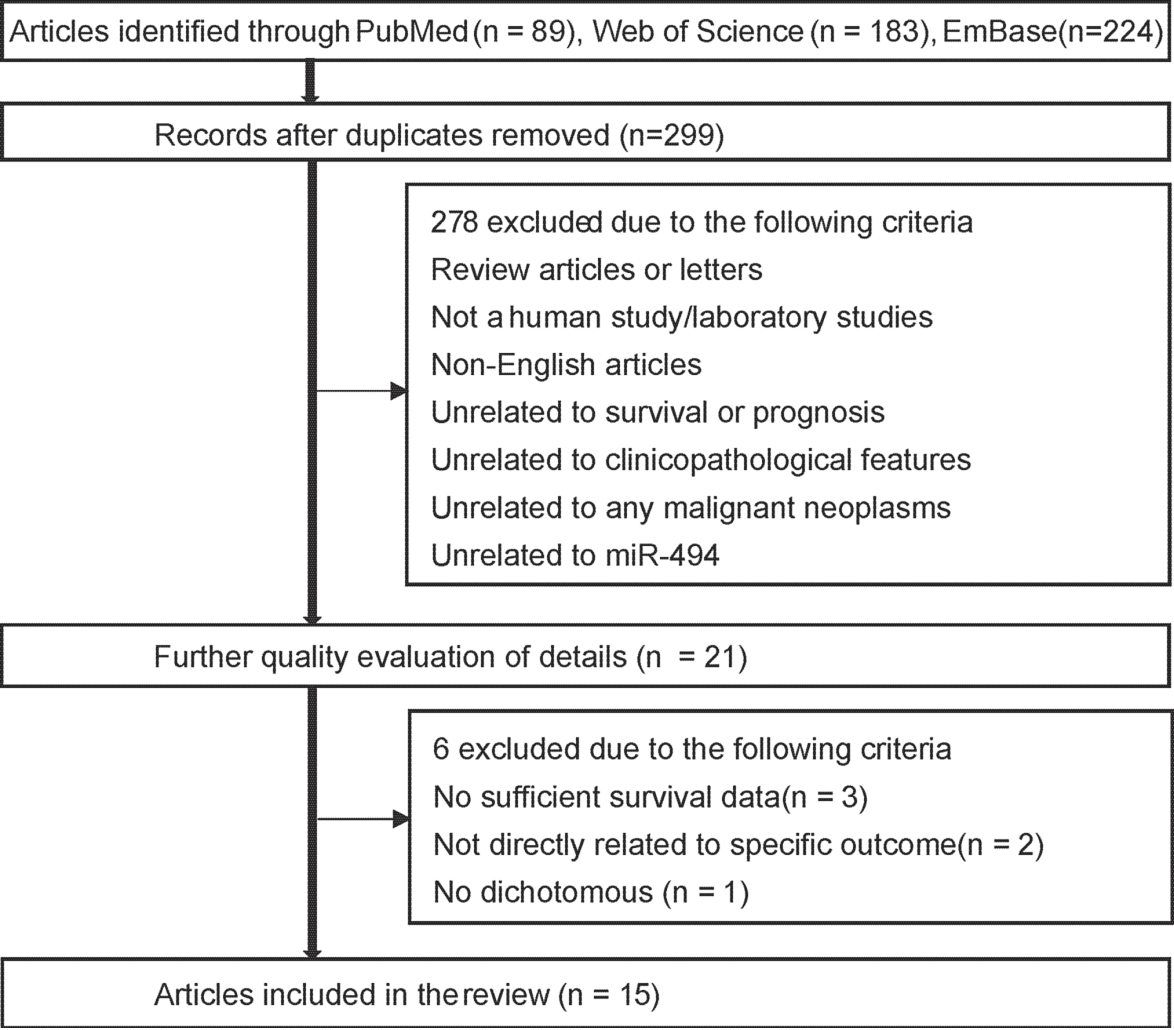
Flow chart of study selection process In the flow chart, there are two wrong words (survival and unrelated), we will send the Figure 1 again.

Using the searching strategy described in MATERIALS AND METHODS, we found 89 articles in PubMed, 183 articles in Web of Science and 224 articles in Embase. After we removed the duplicates, we had 299 articles left. Among the remaining 299 articles, two hundred and seventy-eight articles - which included review, letters, laboratory studies, or articles irrelevant to present research - were ruled out further. After we comprehensively assessed the quality of the remaining 20 articles, fifteen articles were considered eligible for this meta-analysis (Figure 1).

As shown in Table 1, the eligible articles were published from 2014 to 2016 and included a total of 1104 participants with overall survival (OS) data and 440 participants with DFS/PFS/RFS data from China, America, Italy, Iran and Korea. According to their ethnic background, the participants were categorized as Asian or Caucasian. Cancers including pancreatic cancer (PC), non-small cell lung cancer (NSCLC), colorectal cancer (CRC), CC (cervical cancer), chondrosarcoma, gastric cancer (GC), nasopharyngeal carcinoma (NPC), epithelial ovarian cancer (EOC), and head and neck squamous cell carcinoma (HNSCC) were analyzed in this study. Among these studies, three studies (*n* = 216) recruited pancreatic cancer patients, and six studies (*n* = 643) recruited patients with digestive system cancers, which included colorectal cancer, pancreatic cancer, and gastric cancers. All studies measured miR-494 expression in tumor tissue via quantitative real-time polymerase chain reaction (qRT-PCR) or *in situ* hybridization (ISH). Notably, the mean and median value were selected as the cut-off value in most articles.

**Table 1:** Main characteristics of 15 studies after screening

First Author	Year of Publication	Country	Study Design	Type of Cancer	Sample type	Number	Stage	miR-494 Assay	Cut-off Value	Survival Analysis	Source of HR	Maximum Follow-up
Han [21]	2016	China	R	OC	FTT	50	I–IV	qRT-PCR (SYBR Green)	mean value	OS	SC	80 months
Ma [18]	2015	China	R	PC	FTT	99	I–IV	qRT-PCR (SYBR Green)	median value	OS	Reported	60 months
Liu [20]	2015	China	R	PC	Fresh tissue	87	I–IV	qRT-PCR (SYBR Green)	mean value	OS	SC	40 months
Li [19]	2014	USA	R	PC	FFPE	50	I–IV	qRT-PCR (Taqman)	median value	OS	SC	60 months
Wang [27]	2015	China	R	NSCLC	FFPE	92	I–IV	ISH	NR	OS	SC	98 months
Sun [24]	2014	China	R	CRC	Tissue (-)	247	I–IV	qRT-PCR (Taqman)	median value	OS/PFS	Reported	60 months
Li [23]	2015	China	P	CS	Tissue (-)	71	I–IV	qRT-PCR (Taqman)	mean value	OS	Reported	60 months
Dadpay [16]	2015	Iran	R	NPC	FTT	34	I–IV	qRT-PCR (Taqman)	median value	OS	Reported	120 months
Faversani [25]	2016	Italy	R	NSCLC	Tissue (-)	113/57	I–III	qRT-PCR (Taqman)	median value	OS/DFS	Reported	70 months
Yang [26]	2016	China	P	CRC	FTT	104	I–IV	qRT-PCR (Taqman)	3.05	OS/DFS	SC	65 months
Chang [15]	2015	China	P	HNC	Fresh tissue	45	I–IV	qRT-PCR (Taqman)	mean value	OS	SC	65 months
Chen [14]	2016	China	P	AML	Fresh tissue	32	I–IV	qRT-PCR (Taqman)	mean value	OS/RFS	SC	42 months
He [17]	2014	China	P	GC	FTT	56	I–IV	qRT-PCR (Taqman)	0.8 times control	OS	SC	60 months
Lee [22]	2014	Korea	R	CC	FFPE	24	I–IV	qRT-PCR (Taqman)	mean value	OS	SC	135 months
Li [34]	2015	China	R	OC	FTT	40	I–IV	qRT-PCR (Taqman)	mean value	-	-	-

Study design is described as prospective (P) or retrospective (R).

Abbreviations: miR-494,microRNA-494; HR ,Hazard Ratio; NR, not reported; q-PCR, quantitative real-time polymerase chain reaction; OS, overall survival; PC, pancreatic cancer; OC, ovarian cancer; NSCLC, non-small lung cancer; CRC, colorectal carcinoma; AML, Acute myeloid leukemia; NPC, nasopharyngeal carcinoma. GC, gastric cancer. CC, cervical cancer. EOC, Epithelial ovarian cancer. HNSCC, Head and neck squamous cell carcinoma. CS, chondrosarcoma. ISH, *in situ* hybridization. SC, survival cur. PFS, progress free survival. DFS, disease free survival. RFS, recurrence free survival. FFPE, formalin-fixed paraffin-embedded. FTT, Frozen tumor tissue. -, not mentioned.

All the studies investigated the correlation between miR-494 expression and OS; among them, two emphasized disease free survival (DFS) [25–26], only one study focused on recurrence free survival (RFS) [14] and one focused on progress free survival (PFS) [24].

### Correlation between miR-494 expression and prognosis

**Figure 2:**
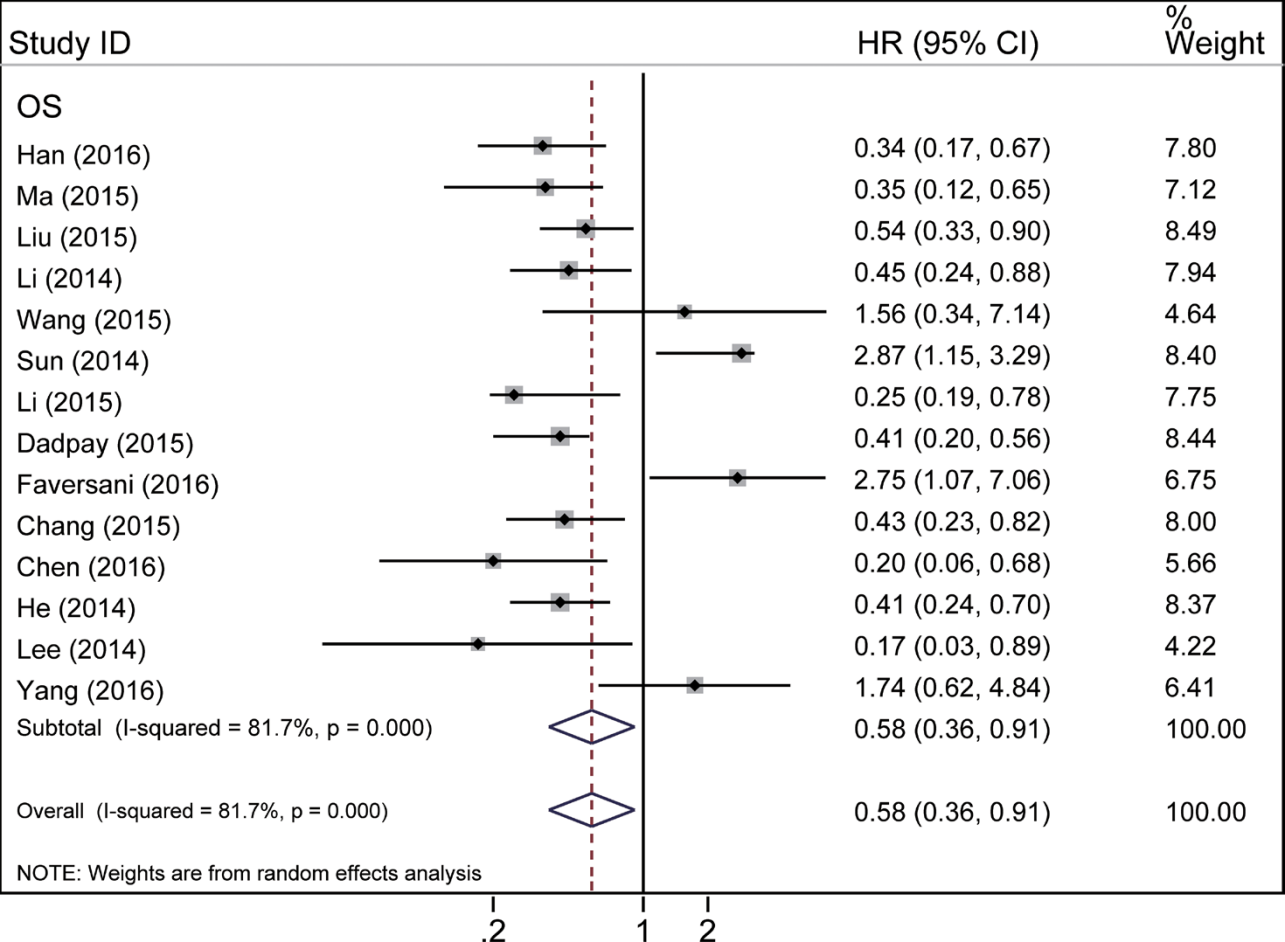
Forest plot of the relationship between miR-494 expression and overall survival in various cancers

Due to the presence of heterogeneity among the studies, all of which related to OS, HR and its 95% CI for OS were pooled via random effects model (*P* < 0.001, *I*^*2*^ = 81.7%) (Figure 2, Table 2). The result revealed that increased expression of miR-494 was an indicator of good OS in various human cancer, with the pooled HR of 0.58 (95% CI: 0.36–0.91) (Figure 2, Table 2).

**Table 2:** Meta-analyisis of overall and subgroup analysis for miR-494 expression and OS in cancers

Category	Studies	HR ( 95% CI )	Model	Heterogeneity	
				I^2^ (%)	*P*
**OS**	14	0.58 (0.36-0.91)	Random	81.7	< 0.001
**Subgroup analysis for OS**					
**Ethnic background**					
Asian	12	0.52 ( 0.32–0.8)	Random	81.6	0.000
Caucasian	2	1.08 ( 0.18–6.33)	Random	89.6	0.002
**Main pathological type**					
Adenocarcinoma	7	0.66 ( 0.34–1.28)	Random	86.4	0.000
Squamous-cell carcinoma	5	0.67 ( 0.29–1.53)	Random	76.1	0.002
**Cancer type**					
Solid tumor	13	0.61 ( 0.38–0.98)	Random	82.4	0.000
Leukemia	1	0.20 ( 0.06–0.68)	Random	-	-
PC	3	0.47 ( 0.33–0.68)	Fixed	0.00	0.681
NSCLC	2	2.35 ( 1.05–5.24)	Fixed	0.00	0.535
CRC	2	2.59 ( 1.62–4.14)	Fixed	0.00	0.394
**Tissue type**					
FTT	5	0.44 (0.33–0.59)	Fixed	48.9	0.098
FFPE	3	0.48 (0.27–0.84)	Fixed	47.9	0.147
Fresh tissue	3	0.45 (0.31–0.66)	Fixed	9.7	0.330
Tissue (-)	3	1.25 (0.24 – 6.43)	Random	93.8	0.000
**MicroRNA assay method**					
qRT-PCR (Taqman)	10	0.60 (0.32–1.11)	Random	86.2	0.000
qRT-PCR (SYBR Green)	3	0.44 (0.30, 0.63)	Fixed	0.0	0.482
ISH	1	1.56 (0.34, 7.15)	Random	-	-
**Anatomy system**					
Respiratory system	3	1.13 (0.28–4.53)	Random	84.9	0.001
Digestive system	6	0.74 (0.36–1.54)	Random	87.3	< 0.001
Reprodution system	2	0.31 (0.16–0.58)	Fixed	0.00	0.450

Abbreviations: miR-494,microRNA-494; HR (Hazard Ratio); CI (Confidence interval); OS, overall survival; PC, pancreatic cancer; NSCLC, non-small cell lung cancer; CRC colorectal carcinoma; FFPE, formalin-fixed paraffin-embedded; FTT, Frozen tumor tissue; “-”, not mentioned.

### Subgroup analysis

**Figure 3:**
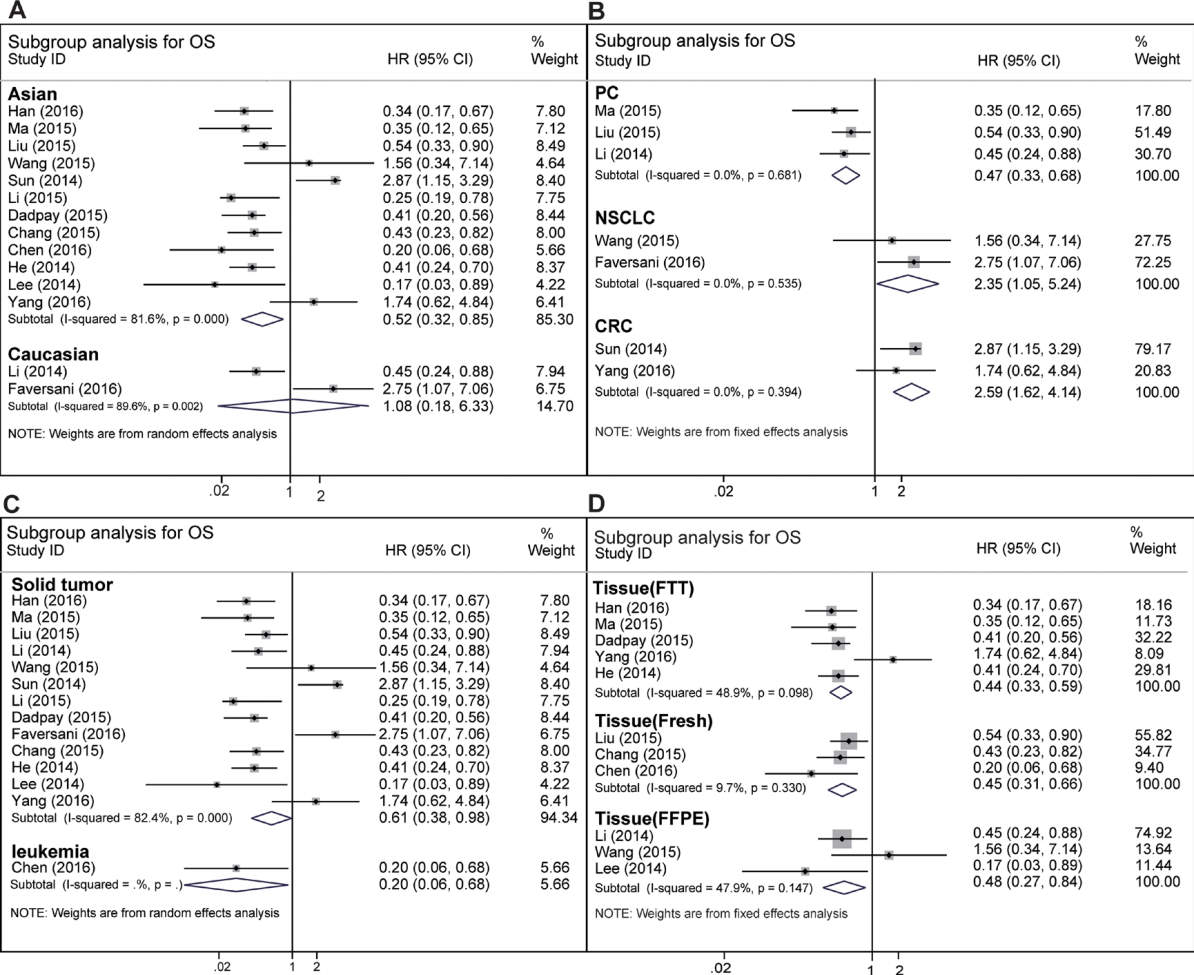
Subgroup analysis of overall survival (**A**) Subgroup analysis of overall survival for Asian or Caucasian cancer patients. (**B**) Subgroup analysis of overall survival for different cancer type. (**C**) Subgroup analysis of overall survival in solid tumor and leukemia. (**D**) subgroup analysis of overall survival in different tissue type (FFPE, FTT, Fresh tissue).

In order to explain the source of heterogeneity in OS, subgroup analysis was performed according to ethnicity (Asian or Caucasian), main pathologic type (Adenocarcinoma or Squamous-cell carcinoma), cancer type, tissue type (Frozen tumor tissue, Fresh tissue, formalin-fxed paraffin-embedded), microRNA assay method (ISH, taqman qRT-PCR, SYBR Green qRT-PCR) and human anatomy system series. To begin with, twelve studies in Asians revealed that elevated expression of miR-494 implies an obviously better OS (HR = 0.52; 95% CI: 0.32–0.85; *I*^*2*^ = 81.6%, *P* = 0.000) via a random-effects model, which was used considering the evident heterogeneity (*I*^*2*^ = 81.6%, *P* < 0.001) among included studies (Figure 3A, Table 2). By merging two studies, we did not find significant correlation between the OS of Caucasians and high miR-494 expression (HR = 1.08; 95% CI: 0.18–6.33; *I*^*2*^ = 89.6%, *P* = 0.002) (Figure 3A, Table 2). As for main pathological type, no significant association was observed between miR-494 expression and squamous cell carcinoma and adenocarcinoma (Supplementary Figure 1A). When the eligible studies were classified in accordance with cancer type, as Figure 3B showed, the Pooled HR of PC was 0.47 (95% CI: 0.33–0.68; *I*^*2*^ = 0.0%, *P* = 0.681), indicating that elevated expression of miR-494 was an indicator of good prognosis in PC. While in NSCLC and CRC (Figure 3B, Table 2), elevated expression of miR-494 was indicator of worse outcome, with the HRs of 2.35 (95% CI: 1.05–5.24; *I*^*2*^ = 0.0%, *P* = 0.535) and 2.59 (95% CI: 1.62–4.14; *I*^*2*^ = 0.0%, *P* = 0.394) respectively. It was obvious that high expression of miR-494 predicted a good OS in the solid tumor and leukemia as per the cancer type (Figure 3C, Table 2). We also found that elevated expression of miR-494 predicted a good OS in Frozen tumor tissue (FTT), formalin-fxed paraffin-embedded (FFPE) and fresh tumor tissue (Figure 3D); but no significant association was found in Tissue which was preserved through unclear method (-) (Supplementary Figure 4). In the subgroup classified as microRNA assay method, we found that elevated expression of miR-494 predicted a good OS in SYBR Green qRT-PCR subgroup; but no significant association was found in taqman qRT-PCR and ISH (Supplementary Figure 5A).

Furthermore, we also categorized the cancer according to the human anatomy system, such as digestive system cancer, respiratory system neoplasms and reproductive system neoplasms. As an obvious heterogeneity existed among the OS of the digestive system and respiratory system neoplasms (Supplementary Figure 1B, Table 2), a random effects model was used to pool the HRs. The results showed that elevated expression of miR-494 would potentially predict good OS for patients with digestive system cancer. However, no potential tendency was found in respiratory system neoplasms, with a pooled HR of 1.13 (95% CI: 0.28–4.53; *I*^*2*^ = 84.9%, *P* = 0.001) (Supplementary Figure 1B, Table 2). We also observed that elevated expression of miR-494 predicted a good OS in reproductive system neoplasms (Supplementary Figure 1C, Table 2), with a pooled HR of 0.31 (95% CI: 0.16–0.58; *I*^*2*^ = 0.0%, *P* = 0.45).

### Sensitivity analysis

**Figure 4:**
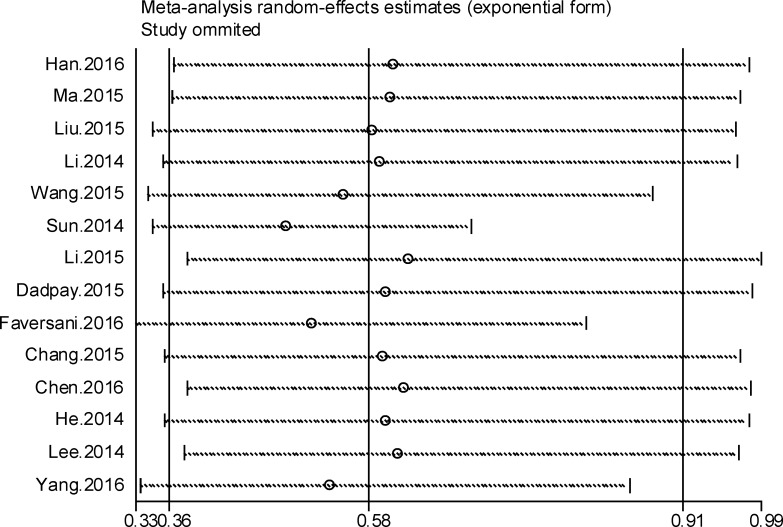
Forest plot of sensitivity analysis for overall survival

In order to test the stability of our results and further seek out the source of heterogeneity, we carried out sensitivity analysis (Figure 4, Supplementary Table 1). In the OS analysis of cancer patients, heterogeneity is significant (*I*^*2*^ = 81.7%, *P* = 0.000). When Sun’s [24], Yang’s [26] and Faversani’s [25] studies were ruled out from the analysis, the heterogeneity for OS became statistically insignificant (*I*^*2*^ = 0%, *P* = 0.538) (Supplementary Figure 2A). According to the same method, we found that Sun’s and Yang’s studies were responsible for the heterogeneity of digestive system cancer subgroup (Supplementary Figure 1B, Supplementary Figure 2B) and Asian subgroup (Figure 3A, Supplementary Figure 2C). In addition, when we iteratively removed studies during the process of sensitivity analysis, the pooled result of remaining studies did not change greatly compared with before, indicating that the entire study was not influenced by any single study (Supplementary Table 1).

### MiR-494 expression and the progress of cancer patients

Given the limited number of studies about the DFS (only two studies) [25–26], PFS (one study) [24] and RFS (one study) [14], we did not combine the HR of PFS, RFS and DFS. We found that elevated expression of miR-494 predicted a shorter DFS in NSCLC (HR = 3.22, 95% CI = 1.02 – 10.2 ) and CRC (HR = 2.5, 95% CI = 1.23 – 5.07). Additonally, the elevated expression of miR-494 predicted a worse PFS in CRC and a good RFS in AML.

### MiR-494 expression and clinicopathological characteristic

Six studies were enrolled in the clinicopathologic analysis. Among them, five studies assessed the association between miR-494 expression and distant metastasis. We combined odds Ratio (OR) via random-effects model due to significant heterogeneity. The result indicates that elevated expression of miR-494 was negatively correlated with distant metastasis (Supplementary Figure 3A), although no statistical significance was found. We also evaluated the connection between overexpression of miR-494 and lymph node metastasis (Supplementary Figure 3B), TNM stage (Supplementary Figure 3C), and tumor differentiation (Supplementary Figure 3D). The pooled OR were 0.53 (95% CI: 0.14–2.02; *I*^*2*^ = 91.5%, *P* = 0.000), 0.79 (95% CI: 0.24 – 2.61; *I*^*2*^ = 88.8%, *P* = 0.000), and 1.06 (95% CI: 0.39–2.87; *I*^*2*^ = 82.3%, *P* = 0.000), respectively, implying that no significant association was revealed between them.

### Assessment of publication bias

**Figure 5:**
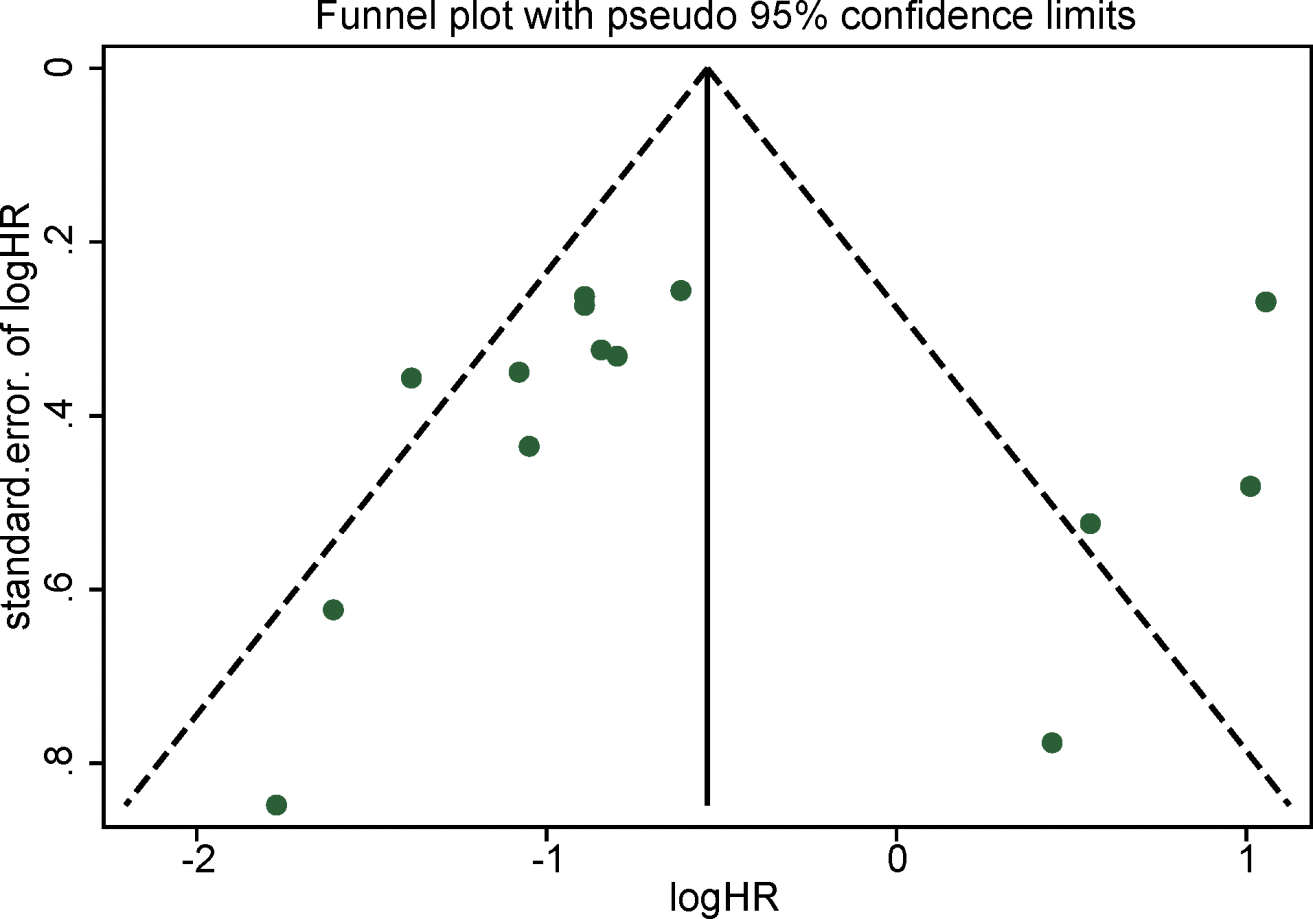
Funnel plot for publication bias in overall survival

Funnel plot, Begg’s test and Egger’s test were employed to assess publication bias of overall survival, the *P* value of which were 0.661 and 0.904 respectively. As funnel plot revealed in Figure 5, no significant asymmetry for pooled overall survival was found.

## DISCUSSION

Variation of biomarker in serum or tissue might exert an important influence in the prognosis of cancer patients, and for this reason, great time and energy has been consumed to establish accurate and reliable biomarkers for cancer patients, which could help doctors make more rational decision. In recent years, microRNAs have been regarded as latent prognosis marker for cancer patients considering their distinct expression profile in the cancer patients’ serum and tissue. Furthermore, the expression of microRNAs are more stable than that of mRNA and it can be readily detected by qRT-PCR. Collectively, miRNA has attracted increasing interest among investigators, especially tumor investigators. Among these available miRNAs, miR-494 has been a particularly attractive one.

To our knowledge, this is the first study investigating the relationship between miR-494 high expression and the prognosis in different kinds of cancer. In our study, increased expression of miR-494 was found to predict good prognosis in cancer patients, with pooled HR of 0.58 (95% CI: 0.36–0.91; *I*^*2*^ = 81.7%, *P* = 0.000) (Figure 2, Table 2). In our meta-analysis, most studies showed that miR-494 target oncogenic gene [14–23] and thus suppress the distant metastasis, lymph node metastasis and tumor proliferation (Table 3). We also carried out subgroup analysis of OS to try to seek out the source of heterogeneity and find out the specific correlation between miR-494 high expression and prognosis of different ethnicity (Asian and Caucasian), main pathologic type, cancer type and human body anatomy system series. When we explored the relationship between the expression level of miR-494 and OS in Asian population with cancers, we observed that elevated expression of miR-494 was significantly related to good OS in the Asian subgroup (Figure 3A, Table 2). However, the prognostic value of miR-494 on non-Asian group remains unclear, with a pooled HR of 1.08 (95% CI: 0.18–6.33; *I*^*2*^ = 89.6%, *P* = 0.002) (Figure 3A, Table 2). Among fourteen studies reporting on OS in ten tumor types, six studies were related to digestive system cancer. Therefore, we performed a subgroup analysis of digestive system cancer. The result did not indicate an obvious association between high miR-494 expression and digestive system cancer (HR = 0.74, 95% CI: 0.36 -1.54) (Supplementary Figure 1B, Table 2), with marked heterogeneity observed in the digestive subgroup (*I*^*2*^ = 87.3%, *P* = 0.000). However, the trend was positive, which indicates that elevated expression of miR-494 would potentially predict good OS for patients with digestive system cancer. We also conducted a subgroup analysis in the cancers of the respiratory system and reproductive system neoplasms. In reproductive system tumor, the results showed that elevated expression of microRNA-494 predicted good OS for cancer patients (Supplementary Figure 1C, Table 2). In contrast, there was no statistically significant associations found in respiratory system cancer (Supplementary Figure 1B, Table 2). Additionally, subgroup analysis was also conducted according to the main pathological types, tissue preservation method and microRNA assay method. There was significant association was found between elevated expression of miR-494 and good OS of FTT subgroup, FFPE subgroup and Fresh tissue (Figure 3D). In the subgroup analysis of microRNA assay method, we found elevated expression of microRNA-494 predicted a good OS in SYBR Green qRT-PCR subgroup (Supplementary Figure 5B). However, no obvious association was found in adenocarcinoma subgroup, squamous-cell carcinoma subgroup (Table 2, Supplementary Figure 1A), unclear tissue preservation subgroup (Supplementary Figure 4), taqman qRT-PCR subgroup and ISH subgroup (Supplementary Figure 5).

**Table 3:** The validated target genes of miR-494 in the eligible studies in this meta-analysis

Study	Year	Type of cancer	Validated target genes
Chen [14]	2016	Acute Myeloid Leukemia	c-Myc
Li [23]	2015	Chondrosarcoma	SOX9
Sun [24]	2014	Colorectal cancer	PTEN
Wang [27]	2015	Non-small cell lung cancer	PTEN
Li [34]	2015	Epithelial ovarian carcinoma	IGF1R
Han [21]	2016	Ovarian cancer	CUL4A
Chang [15]	2015	Head and neck squamous cell carcinomas	Bmi 1, ADAM10
Liu [20]	2015	Pancreatic cancer	c-Myc, SIRT1
Li [19]	2014	Pancreatic cancer	FOXM 1
He [17]	2014	Gastric carcinoma	c-Myc

Abbreviations: SOX9, SRY-related high mobility group-Box gene 9; PTEN, phosphatase and tensin homolog deleted on chromosome ten; IGF1R, insulin-like growth factor 1 receptor; CUL4A, Cullin 4A; Bmi 1, B-cell-specific moloney leukemia virus insert site 1; ADAM10, A disintegrin and metalloproteinase 10; SIRT1, Sirtuin Type 1; Foxm1, Forkhead box protein M1.

To eliminate the effect of different mechanism which was induced by the process of tumor formation and tumor progression among various cancers, we conducted a subgroup analysis according to the cancer type. It was noted that increased expression of miR-494 was strongly correlated with good OS (HR = 0.47, 95% CI: 0.33–0.68; *I*^*2*^ = 0.0%, *P* = 0.681) in PC (Figure 3B, Table 2). It may be because miR-494 high expression suppressed the tumor cell monolayer proliferation, colony formation and tumorsphere formation by negatively regulating the expression of FOXM1. Accordingly, the expression level of miR-494 in tissue or serum was regarded as a promising biomarker for prognosis of PC [19]. However, we draw an entirely contrary conclusion to most studies in NSCLC (Figure 3B, Table 2) subgroup where increased expression of miR-494 predicted obvious worse survival rate. Giulia-Romano found that the increased expression of miR-494 lead to the Tumor necrosis factor Related Apoptosis-Inducing Ligand (TRAIL) resistance in NSCLC via down regulation of BCL2L11, which may inhibit the apoptosis and promote the tumorigenicity of tumor cells [28]. Researchers, therefore, consider miR-494 to play a significant part in promoting tumor formation and death of NSCLC. As shown in Figure 3B, elevated expression of miR-494 also predicted a worse outcome in CRC. It can be hypothesized that elevated expression of miR-494 promotes the invasion and migration of tumor cells by targeting PTEN, which was one of the most frequently mutated tumor suppressor genes in human cancer and may result in shorter survival rate in CRC [24]. We also find that elevated expression of miR-494 predicted a shorter DFS in NSCLC (HR = 3.22, 95% CI = 1.02 – 10.2) and CRC (HR = 2.5, 95% CI = 1.23 – 5.07). It was obvious that high expression of miR-494 predicted a good OS in the solid tumor and leukemia as per the cancer type (Figure 3C, Table 2). We also observed that increased expression of miR-494 forecast good OS in OC, HNSCC, NPC, AML, GC and CC. Through the subgroup analysis, the heterogeneity of some subgroup analysis remains huge still, so the subgroup analysis could not elucidate the source of heterogeneity clearly. In order to further seek out the source of heterogeneity, sensitivity analysis was conducted. According to the sensitivity analysis, Sun’s, Yang’s and Faversani’s studies lay behind the source of heterogeneity for OS analysis. When we removed these studies from current studies, the heterogeneity became insignificant (*I*^*2*^ = 0.0%, *P* = 0.538) (Supplementary Figure 2A). Compared with most studies in this meta-analysis, these studies showed the opposite outcome that miR-494 high expression predicted a worse outcome of cancer, which may cause fairly large heterogeneity in current research. Using the same method, we conducted a sensitivity analysis in the Asian group and digestive group, and we found that the Sun’s and Yang’s study were responsible for the heterogeneity of this two groups. When we removed these studies in these two groups, the heterogeneity became insignificant (Supplementary Figure 2C, Supplementary Figure 2B). When we removed one study at a time from all eligible studies during the process of sensitivity analysis, no single study obviously affected the entire pooled result (Figure 4, Supplementary Table 1), which indicates that the result is stable. Additonally, the elevated expression of miR-494 predicted a worse PFS in CRC and a good RFS in AML.

Furthermore, we conducted an analysis between the expression of miR-494 and clinicopathologic factors. Elevated expression of miR-494 was negatively associated with distant metastasis, which suggested that elevated expression of miR-494 might suppress distant metastasis of cancer patients (Supplementary Figure 3A), although no obvious statistical significance was found between them. Meanwhile, no statistically significant relationship were found between the high expression of miR-494 and TNM stage (Supplementary Figure 3C), tumor differentiation (Supplementary Figure 3D), and lymph node metastasis (Supplementary Figure 3B). As the number of studies about the association between the expression of miR-494 and clinicopathologic factors was limited, more related articles will be needed to conduct further investigation.

It’s interesting that high expression of miR-494 predicted a good OS of cancer patients, but no significant association was found in clinicopathologic characteristics. Additionally, the prognostic value of miR-494 in Asian and Caucasian are not consistent. In cancer, miR-494 act as tumor suppressors or promoter depending on their target gene. If the target gene of miR-494 involved in cell differentiation or as tumor suppressors, microRNA bind to complementary sequences of mRNA at the 3’-untranslated region and lead to mRNA degradation or translational repression [4, 5], the miR-494 act as oncogenes [35]. Similarly, microRNAs act as tumor suppressors [35] when they downregulate different proteins with oncogenic activity. In our meta-analysis, most studies showed that miR-494 act as tumor suppressors and their high expression predicted a good OS [14–23]. While a few studies reported inconsistent results [24–27], indicating miR-494 maybe a tumor promotor. The reason is that miR-494 regulate different target genes in different cancer type (Table 3). The limited research of Caucasicans (two studies) and clinicopathologic characteristics (six studies) make the result easily affected, which means any single study might affect the entire result. From the Supplementary Figure 3 we could find that the result was affected most by Sun’s [24] and Wang’s [27] study, which target same carcinostatic genes PTEN (Table 3) and downregulate PTEN, thus promoting the progress of tumor. Besides that, the limit of language (only in English) makes it impossible to get more studies. On the other hand, the cancer type varies in different subgroup (Asian and Caucasians, different clinicopathological charactersitic), but miR-494 downregulate different target genes in different cancer type (Table 3), thus result in the different prognosis and affected the conclusion in subgroup. Although no significant association was found between miR-494 expression and clinicopathological characteristic (Supplementary Figure 3), the trend of most result were positive (three out of four), which indicates that high expression of miR-494 negatively relate to distant metastasis, lymph node metastasis and TNM stage. So It is reasonable for us to get these conclusions, but we should treat the conclusion cautiouly before we apply it in some specific tumor type and specific subgroup, unless we have demonstrated it in large number of studies.

Although great efforts have been made to minimize errors, several limits persist in this article. First, the HRs of some articles were extracted and calculated from survival curves, which might deviate from the original values, even slightly. Second, we don’t have access to miR-494 expression data of global populations, which makes it hard to set a standard cut-off. so the cut-off values of miR-494 expression are not consistent among included studies, which makes it hard to set a consistant criteria for high expression and makes our conclusion less persuasive. Third, most sample included in this meta-analysis were tissue, little in serum or urine. Compared with tissue, circulating biomarkers or urine are easier to be accepted by patients and more convenient for permanent monitoring. Forth, because of the limited research for each cancer type, more related studies will be needed to confirm the correlation between miR-494 expression level and prognosis of various types of cancer. Fifth, there exists relatively large heterogeneity in this meta-analysis (*I*^*2*^ = 81.7%, *P* = 0.000). According to the sensitivity analysis, the heterogeneity mainly came from Sun’s, Yang’s, and Faversani’s study. When we removed them from this meta-analysis, the adjusted HR was 0.40, and the heterogeneity become insignificant (*I*^*2*^ = 0.0%, *P* = 0.538). Besides, different detection methods, sample sources, preservation of tumor tissue, microRNA assay method and cut-off values may also have affected the effectiveness of miR-494 as a predictive biomarker, causing relatively large heterogeneity. Additionally, a panel of miRNAs may be a stronger predictor for prognosis than a single miRNA, which ought to be relatively cheap and have high specificity and sensitivity. In the current research, neither Begg’s test nor Egger’s test showed significant publication bias for overall survival.

Despite the limits described above, our study clearly demonstrates that elevated expression of miR-494 is correlated with good overall survival in cancer patients, especially in Asian people and pancreatic cancer. Whereas in NSCLC and CRC, elevated expression of miR-494 predicted a short overall survival time. Multicenter, and large sample studies are still lacking to further validate these conclusions, and more clinical studies ought to be carried out before the application of the prognostic value of miR-494 in cancer, especially for any single type of cancer.

## MATERIALS AND METHODS

### Search strategy

We comprehensively searched Embase, PubMed, Web of Science databases from 1966 to April 1st, 2017.The literature was published during the period of time, grey literature was not found during our meta-analysis. The keywords employed in searching were “mir-494 OR microrna-494 OR mirna-494 OR hsa-mir-494” AND “tumor OR cancer OR carcinoma OR neoplasm”. To get some raw data which was not mentioned in the articles, we contacted some of the authors to get a more accurate result. Relevant references of review articles about miR-494 were also searched by hand and carefully read by us to obtain more studies. In addition, we separately conducted the search and articles inclusion based on a common set of criteria, and we settled our divergence in opinion by discussion among ourselves.

### Inclusion and exclusion criteria

We conducted this meta-analysis on the basis of PRISMA (Preferred Reporting Items for Systematic Reviews and Meta-Analysis) statement. Studies were enrolled in this research if they satisfy the following conditions: (i) patients involved in this meta-analysis must be diagnosed with cancer via pathology; (ii) The method for detecting the expression of miR-494 must be q-PCR or ISH; (iii) The correlation between miR-494 expression and prognosis or clinicopathological features was investigated; (iv) The HR and its 95% CI for OS on the basis of miR-494 expression level were readily available or could be calculated indirectly; (v) When any single study patient sample source was used in several studies, we chose the most representative and most accurate study to avoid unnecessary cohort overlapping. In addition, studies that have already satisfied the abovementioned inclusion requirements were further ruled out if they had any of the following flaws: (i) duplicated articles or data; (ii) not human studies; (iii) review articles or letters; (iv) lack of sufficient data or information to get HR; (v) articles not written in English.

### Quality assessment

We systematically assessed the quality of eligible studies per the critical checklist of the Dutch Cochrane Centre, which was proposed by MOOSE [29, 30]. The key points of the quality assessment contained clear description of the following: (i) the main ethnic background and country; (ii) the cancer type; (iii) outcome assessment; (iv) the detection method of miR-494; (v) cut-off value; (vi) adequate follow-up time. We excluded articles which missed any of the aforementioned key points.

### Data extraction and quality assessment

The full texts of eligible studies were carefully read by us, then we extracted the following data independently: (i) the first author (ii) publication year; (iii) characteristics of the studies, which comprise the patients’ nationality, sample size, tumor type, and clinicopathological characteristics; (iv) the assay method and cut-off value of miR-494; (v) HRs of miR-494 expression for OS, recurrence-free survival (RFS), disease-free survival (DFS), progression-free survival (PFS); (vi) if the HR for OS, PFS, DFS, and RFS were calculated by both univariate and multivariate analyses, the latter was our first choice, given that these results were adjusted for confounding factors. If a study did not report the HR directly, the method described by Parmar et al. [31] and Tierney et al. [32] was employed to estimate HR and their corresponding 95% CI. We recovered the data of Kaplan-Meier curves via the Engauge Digitizer and calculated the HR and its 95% CI according to the method described above, and we repeated this process three times to reduce variability. We resolved divergence by discussion among ourselves, until consensus was reached regarding the extraction and interpretation of all data.

### Statistical analysis

All the HRs and their 95% CIs were combined to evaluate the effect of miR-494 high expression to prognosis. Generally, if the pooled HR < 1 and their 95% CI did not overlap the invalid line in the forest, the high expression of miR-494 predicted a good OS. If the 95% CI overlapped the invalid line, the combined HR remained insignificant. Otherwise, the combined HR predicted a worse OS. The heterogeneity of pooled result was checked via Cochran’s *Q* test and Higgins’ I-squared, and it was defined as *P* < 0.1 or *I*^*2*^ > 50%. If *P* > 0.1 and *I*^*2*^ < 50%, we ignored the influence of heterogeneity and a fixed effects model was employed to pool the overall result, otherwise the random effects model was employed. The potential publication bias was assessed by funnel plot, Begg's and Egger’s test (if *P* < 0.05, publication bias was statistically significant) [33]. Sensitivity analysis, by means of iteratively removing each study, was employed to evaluate the stability of the results. A *p*-value less than 0.05 was considered to be statistically significant.

## SUPPLEMENTARY MATERIALS FIGURES AND TABLE



## Abbreviations

miRNA-494/miR-494: microRNA-494
HR: hazard ratio
CI: confidence interval
NR: not reported
qRT-PCR: quantitative real-time polymerase chain reaction
OS: overall survival
PC: pancreatic cancer
OC: ovarian cancer
NSCLC: non-small lung cancer
CRC: colorectal carcinoma
AML: Acute myeloid leukemia
NPC: nasopharyngeal carcinoma
GC: gastric cancer
CC: cervical cancer
EOC: Epithelial ovarian cancer
HNSCC: Head and neck squamous cell carcinoma
CS: chondrosarcoma
ISH: *in situ* hybridization
SC: survival curve
PFS: progress free survival
DFS: disease free survival
RFS: recurrence free survival
FFPE: formalin-fixed paraffin-embedded
FTT: Frozen tumor tissue
“-”: not mentioned
