# Supplementary material for: Prognostic and clinicopathological significance of microRNA-494 overexpression in cancers: a meta-analysis
Source: Oncotarget. 2017 Nov 3;9(1):1279–90. doi: 10.18632/oncotarget.22633 (PMC5787438; doi:10.18632/oncotarget.22633)
Supplement: Supplementary file 1 [file oncotarget-09-1279-s001.pdf]

Prognostic and clinicopathological significance of microRNA-494 overexpression in cancers: a meta-analysis

SUPPLEMENTARY MATERIALS

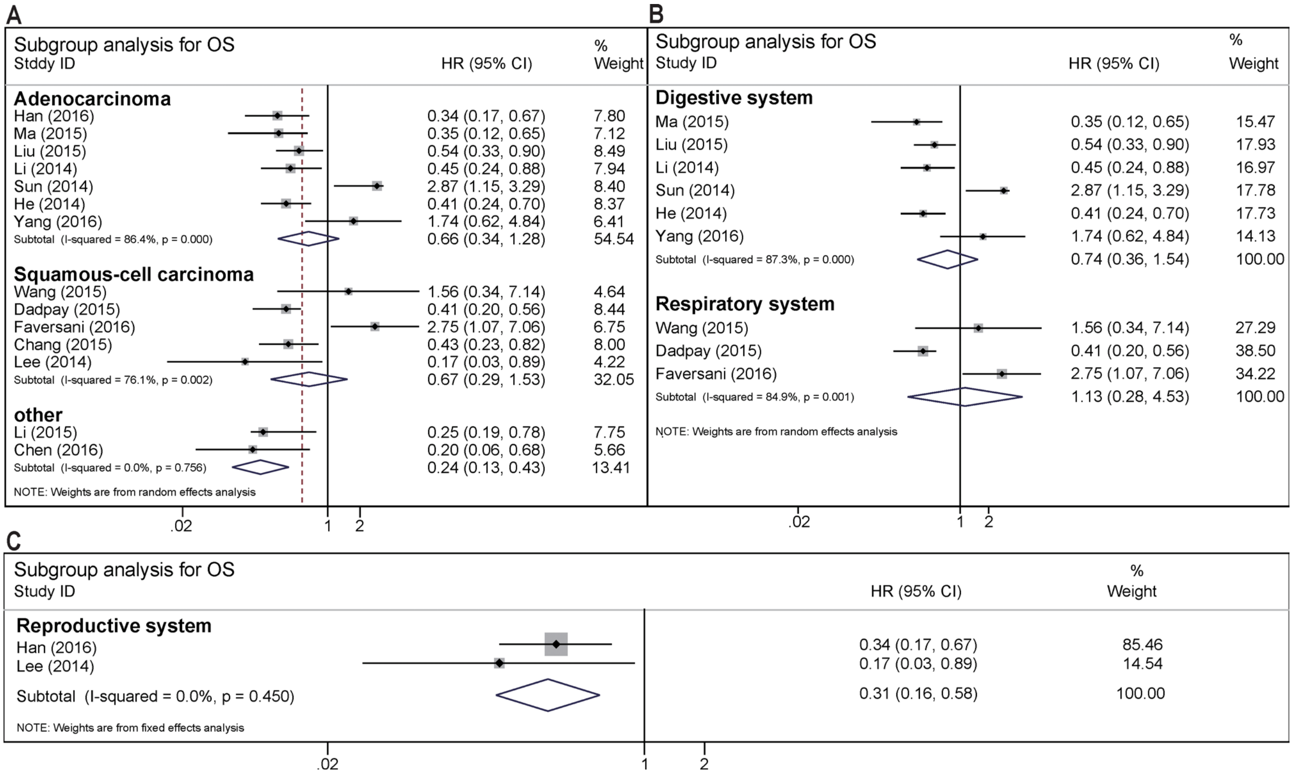

Supplementary Figure 1: Forest plot of subgroup overall survival analysis for (**A**) main pathological type; (**B**) digestive and respiratory system; (**C**) reproductive system.

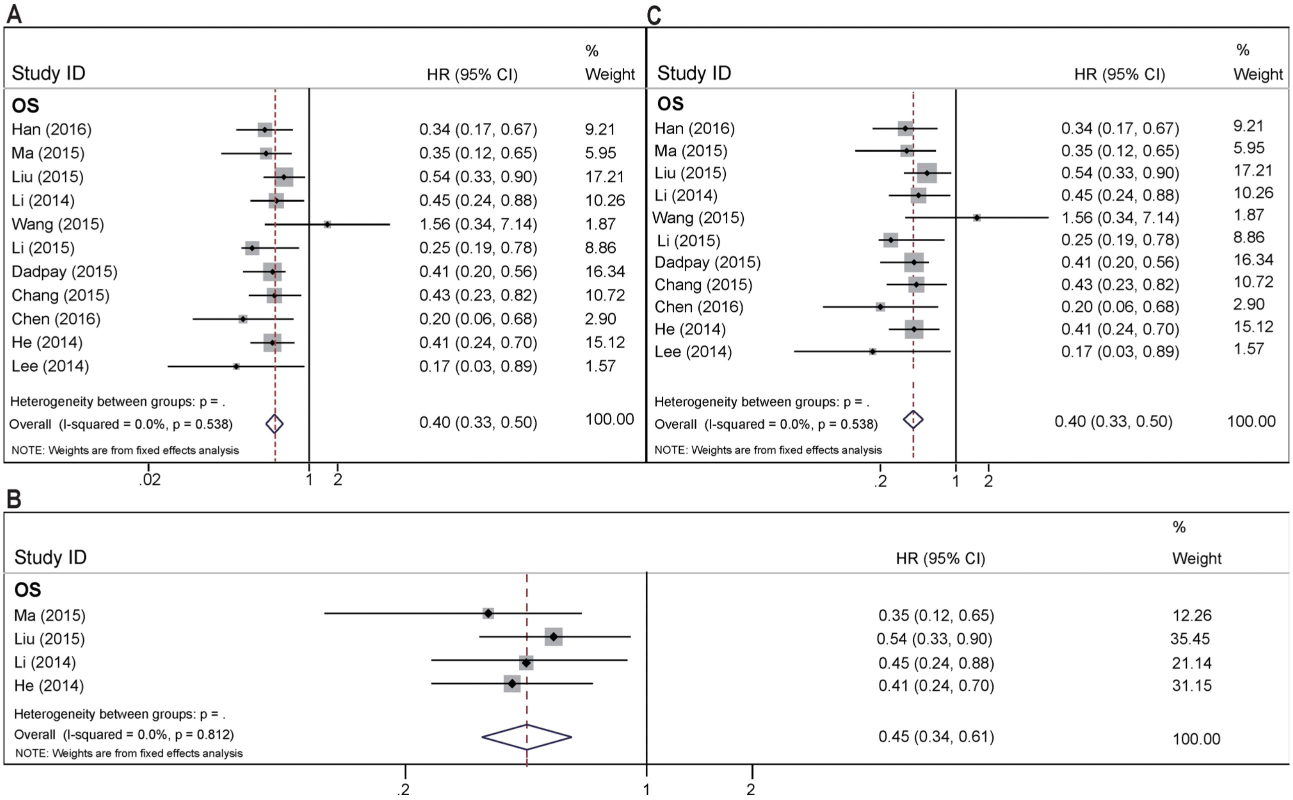

Supplementary Figure 2: The forest plot of remaining studies when we removed the study which caused relatively large heterogeneity. (**A**) When we removed the Sun's, Yang's, and Faversani's study from overall survival analysis, the forest plot of remaining studies. (**B**) When we removed the Sun's and Yang's studies from digsetive subgroup, the forest plot of remaining studies. (**C**) When we removed the Sun's and Yang's studies from Asian subgroup, the forest plot of remaining studies.

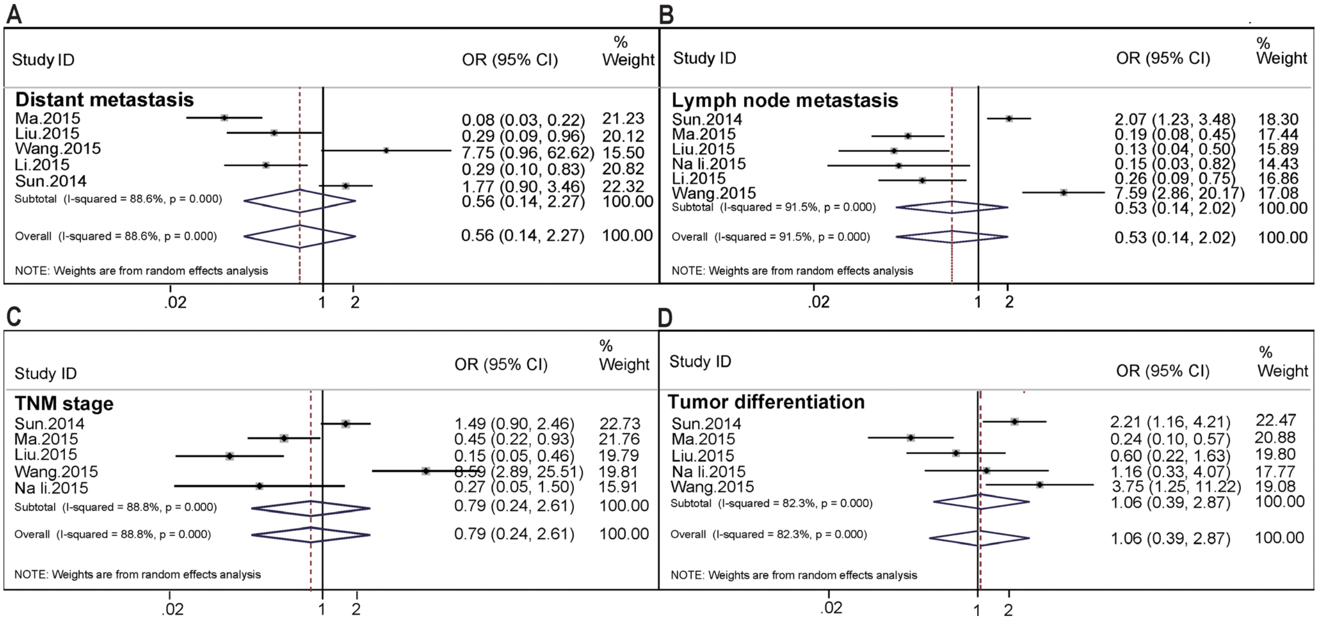

Supplementary Figure 3: Forest plot of the relationship between miR-494 high expression and clinicopathological characteristic. (**A**) Forest plot of the relationship between miR-494 high expression and distant metastasis. (**B**) Forest plot of the relationship between miR-494 high expression and lymphatic metastasis. (**C**) Forest plot of the relationship between miR-494 high expression and TNM stage. (**D**) Forest plot of the relationship between miR-494 high expression and tumor differentiation.

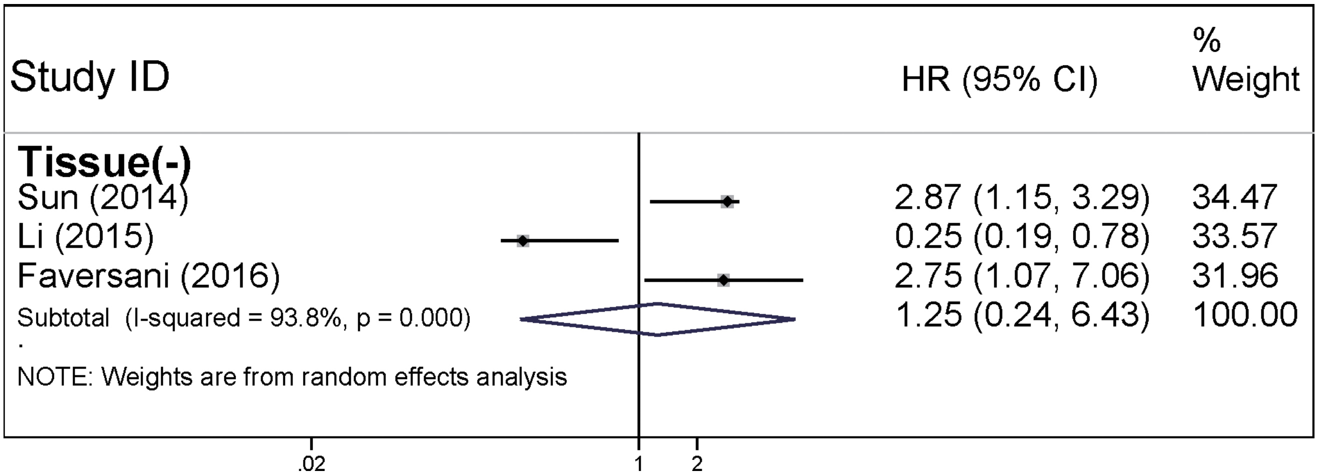

Supplementary Figure 4: Forest plot of subgroup overall survival analysis in tissue preservation method (−). "(−)" means not mention the preservation method of tumor tissue.

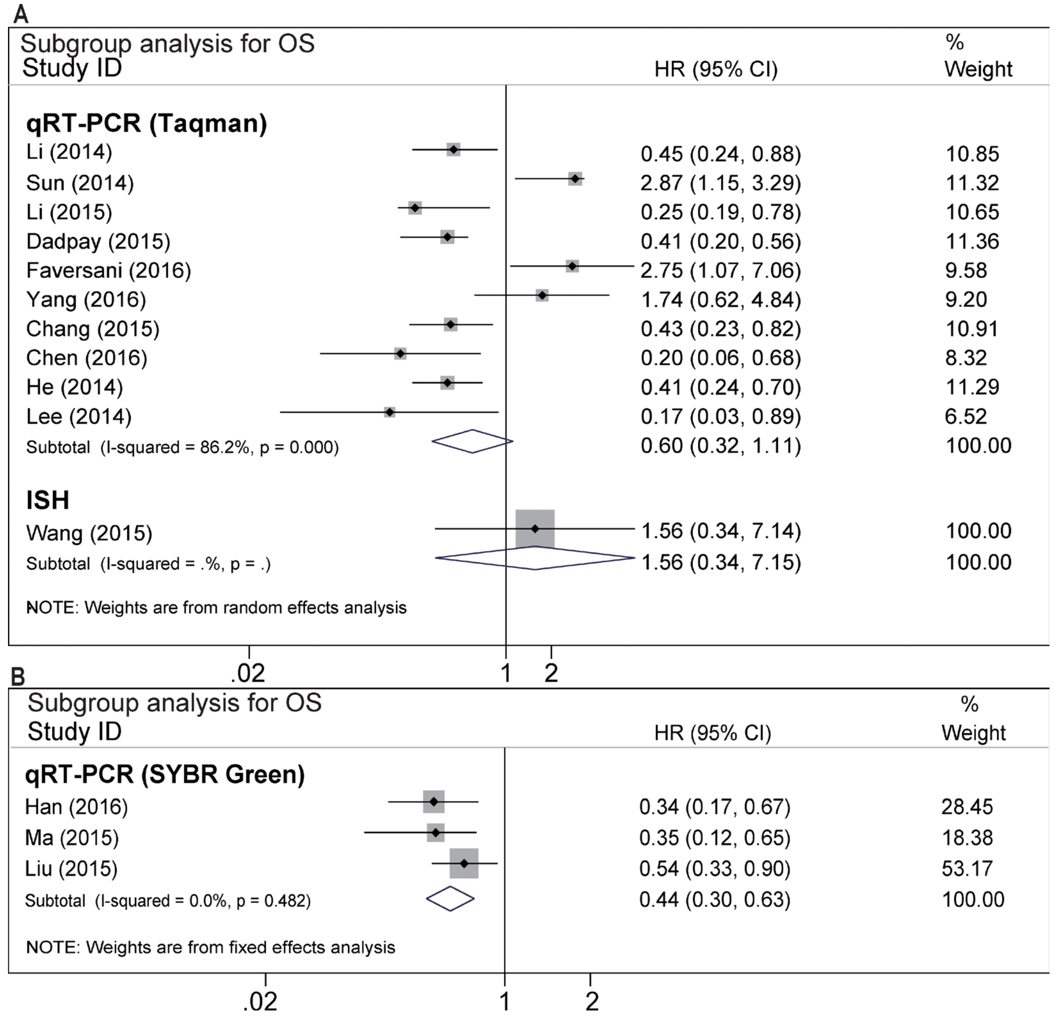

Supplementary Figure 5: Subgroup analysis of overall survival in different miR-494 assay method. (**A**) Forest plot of the relationship between miR-494 high expression and overall survival analysis in Taqman qRT-PCR subgroup and ISH subgroup. (**B**) Forest plot of the relationship between miR-494 high expression and overall survival analysis in SYBR Green qRT-PCR subgroup.

Supplementary Table 1: Sensitivity analysis for overall survival

Study ommited	HR	95% CI	Heterogeneity after omitted
Han 2016 [21]	0.60	0.37–0.98	$I^2 = 82.5\%$
Ma 2015 [18]	0.60	0.37–0.97	$I^2 = 82.8\%$
Liu.2015 [20]	0.58	0.35–0.97	$I^2 = 83.1\%$
Li 2014 [19]	0.59	0.36–0.97	$I^2 = 82.9\%$
Wang 2015 [27]	0.55	0.34–0.88	$I^2 = 82.7\%$
Sun 2014 [24]	0.49	0.35–0.68	$I^2 = 61.6\%$
Li 2015 [23]	0.62	0.38–0.99	$I^2 = 82\%$
Dadpay 2015 [16]	0.59	0.36–0.98	$I^2 = 82.6\%$
Faversani 2016 [25]	0.51	0.33–0.81	$I^2 = 80.1\%$
Chang 2015 [15]	0.59	0.36–0.97	$I^2 = 82.9\%$
Chen 2016 [14]	0.61	0.38–0.98	$I^2 = 82.4\%$
He 2014 [17]	0.59	0.36–0.98	$I^2 = 82.6\%$
Lee 2014 [22]	0.61	0.38–0.97	$I^2 = 82.6\%$
Yang 2016 [26]	0.53	0.33–0.85	$I^2 = 81.5\%$
Combined	0.58	0.36–0.91	$I^2 = 81.70\%$
